# The Initial Stage in Oxidation of ZrNiSn (Half Heusler) Alloy by Oxygen

**DOI:** 10.3390/ma12091509

**Published:** 2019-05-09

**Authors:** Oshrat Appel, Gil Breuer, Shai Cohen, Ofer Beeri, Theodora Kyratsi, Yaniv Gelbstein, Shimon Zalkind

**Affiliations:** 1 Department of Materials Engineering, Ben-Gurion University of the Negev, POB 653, Beer-Sheva 84105, Israel; gil.breuer@gmail.com (G.B.); yanivge@bgu.ac.il (Y.G.); 2 Nuclear Research Centre-Negev, POB 9001, Beer-Sheva 84190, Israel; scking1@gmail.com (S.C.); ofer.beeri@gmail.com (O.B.); 3 Department of Mechanical and Manufacturing Engineering, University of Cyprus, Kallipoleos 75, Nicosia 1678, Cyprus; kyratsi@ucy.ac.cy

**Keywords:** ZrNiSn, half-Heusler, thermoelectric, segregation, surface oxidation, oxygen, XPS

## Abstract

The MNiSn (M = Ti; Zr; Hf); half-Heusler semiconducting alloys have a high potential for use as *n*-type thermoelectric materials at elevated temperatures (~1000 K). The alloys’ durability is crucial for their commercial handling and use, and therefore it is required to characterize their surface oxidation behavior and stability at the working temperature. X-ray photoelectron spectroscopy was utilized to study the surface composition and oxidation of the ZrNiSn alloy at room and elevated temperatures. It was found that during heating in a vacuum, Sn segregates to the surface in order to reduce the surface energy. Exposing the alloy to oxygen resulted mainly in the oxidation of the zirconium to ZrO_2_, as well as some minor oxidation of Sn. At room temperature, the oxidation to ZrO_2_ was accompanied by the formation of a thin ZrO layer at the metal-oxide interface. In contrast to TiNiSn, where most of the oxide was formed on the surface due to oxygen-enhanced segregation of Ti, and in the case of ZrNiSn, the formed oxide layer was thinner. Part of the oxide is formed due to Zr segregation to the surface, and in part due to oxygen dissolved into the alloy.

## 1. Introduction

Thermoelectric applications are being widely investigated, mainly because of their main purpose of decreasing the dependence on conventional fossil fuels and reducing harmful emissions [1]. Half-Heusler (HH) is a family of intermetallic semiconducting compounds exhibiting high Seebeck coefficient and low electrical resistivity values, along with superior mechanical properties, making them thermoelectrically attractive [2]. Their stability at high temperature makes them good candidates for thermoelectric modules that operate up to 1000 K. The compounds crystallize in the cubic MgAgAs-type structure ($F\bar{4}3m$), and can be derived from the tetrahedral ZnS-type structure by filling the octahedral lattice sites [3]. One of the most investigated HH compounds is the *n*-type MNiSn-based family, where M stands for Ti, Zr or/and Hf [4,5,6], which are widely available, inexpensive and nontoxic [7]. 

For practical applications, it is crucial to take into account other considerations also, such as the environmental stability at working conditions. Although numerous studies have been conducted regarding the alloys’ metallurgical and transport properties, only few studies can be found on their corrosion behavior and environmental stability, especially at the high operation temperatures, which are a key factor for the commercial utilization of the alloys [8,9,10]. 

In our previous study [11], the surface properties of the TiNiSn alloy and their interaction with oxygen and water vapor at room temperature (RT) and 1000 K were characterized for the first time. It was found that during the heating of the sample in a vacuum, Sn readily segregates to the surface due to the considerably lower surface energy of the Sn compared to the Ni and Ti. During oxidation it was found that Ti segregates to the surface and oxidizes, forming, at 1000 K, a titanium oxide layer with a variety of oxidation states, from Ti^+2^ at the alloy-oxide interface, and up to Ti^+4^ at the outer region. Some mild oxidation of Sn was also observed. Water was found to be less reactive with the sample compared to oxygen. The oxidation tendency of the alloy was found to be in consensus with the enthalpy of formation of the oxides’ components. 

As part of the motivation to map the surface properties and the oxidation behavior of other MNiSn HH thermoelectric alloys at the expected operation temperature, in the present work, temperature effects on the surface composition and the initial oxidation behavior of ZrNiSn alloy at RT and 1000 K were studied, utilizing X-ray photoelectron spectroscopy (XPS). The results are compared to those recently reported [11] for the TiNiSn alloy.

## 2. Experimental

**Figure 1:**
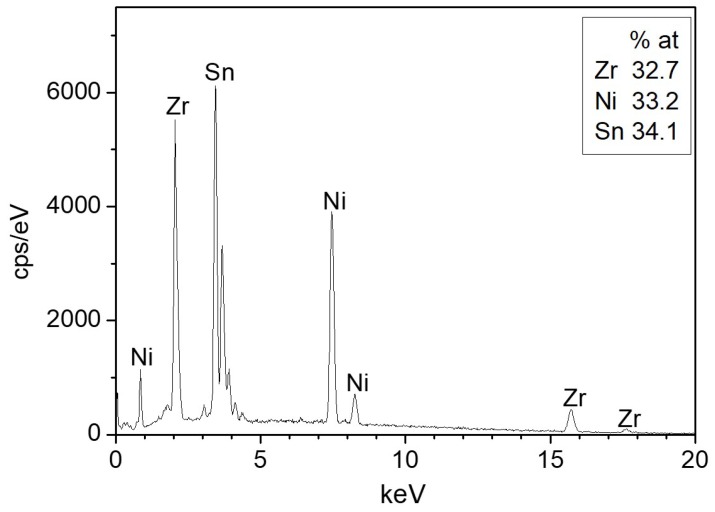
Representative energy dispersive spectroscopy (EDS) spectrum of the sample surface.

**Figure 2:**
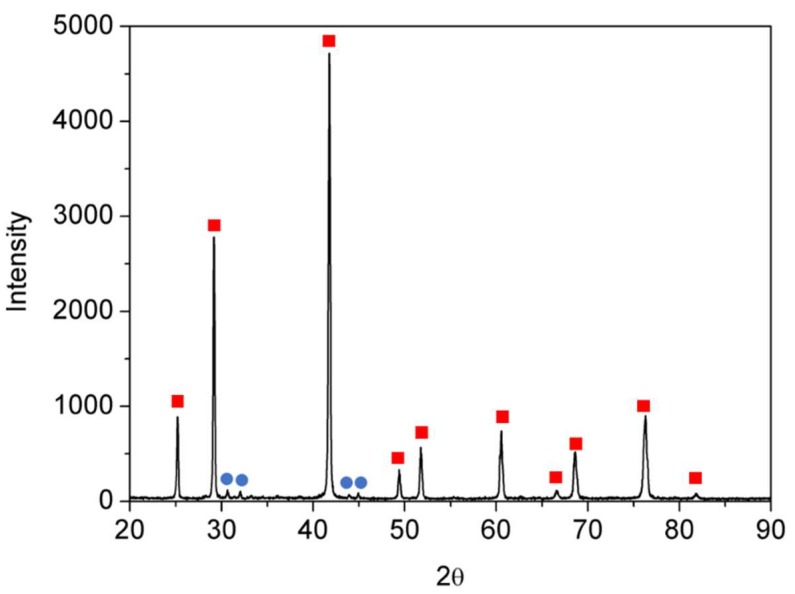
X-ray diffractogram (XRD) spectra of the synthesized sample. Red squares indicate the HH phase and the blue circles, the minority Sn phase.

The ZrNiSn alloy was prepared from high purity components by arc melting, under Ar atmosphere. The alloy was re-melted five times, and the composition and homogeneity were verified by scanning electron microscopy (SEM, JSM5600, JEOL Ltd., Tokyo, Japan) and energy dispersive spectroscopy (EDS, Thermo Fisher Scientific, Waltham, MA, USA), as shown in Figure 1. The measured stoichiometry by the EDS was Zr_0.98_Ni_1.00_Sn_1.02_. Figure 2 presents an X-ray diffractogram (XRD, Rigaku DMAX 2100 powder diffractometer, Tokyo, Japan) of the alloy, revealing its half-Heusler structure ($F\bar{4}3m$) and a small amount of residual Sn, as expected from the EDS quasi-quantitative chemical analysis.

A ~6 mm diameter and 1 mm thick sample was cut out from the casting, and was gradually polished using abrasive papers and diamond paste down to 1 µm. The sample was attached to two Ta wires, which enabled heating by driving an electric current through it. The sample’s temperature was monitored by a chromel-alumel thermocouple, spot-welded to the sample’s edge. 

The experiments were performed in an ultra-high-vacuum (UHV) system, pumped by turbo-molecular and titanium sublimation pumps to a base pressure of ~2 × 10^−10^ Torr. The system contains standard surface analysis instrumentation for X-ray photoelectron spectroscopy (XPS, ESCALAB 250, Thermo Fisher Scientific, Waltham, MA, USA), and a differentially pumped rastered Ar^+^ gun for surface cleaning. The XPS measurements were performed using an Al-anode X-ray source (hν = 1486.6 eV). The survey scans and the high-resolution Ni 2p and Sn 3d peaks were measured with spectrometer pass energy of 50 eV, while the Zr 3d and O 1s peaks were measured with 20 eV pass energy. The Au 4f_7/2_ peak at binding energy (BE) of 84 ± 0.1 eV and Sn 3d_5/2_ at 485 ± 0.1 eV, taken from clean pure reference samples, at similar parameters as of the alloy, were used to verify the energy calibration of the spectrometer.

The oxidation experiments were performed by backfilling the vacuum chamber with O_2_ (99.999%) via a leak valve to a pressure of 1 × 10^−6^ Torr. The exposure is given in Langmuir (1 L = 1 × 10^−6^ Torr × 1 sec.).

The spectra were analyzed using the CasaXPS software (2.3.15, Casa Software Ltd., Cheshire SK9 6BN, Devon, UK). The XPS data analysis of the high resolution spectra was performed using Shirley background and the constraints on energy locations of the different oxidation states and split-orbit area ratio are detailed in [12,13,14,15,16]. A mixture of Gaussian-Lorentzian line shape GL (30) was used for the oxides fitting, while for the metallic core lines a slightly asymmetric shape in the form of LA(α,β,m) was used. 

## 3. Results and Discussion

### 3.1. Surface Characterization and Segregation

**Figure 3:**
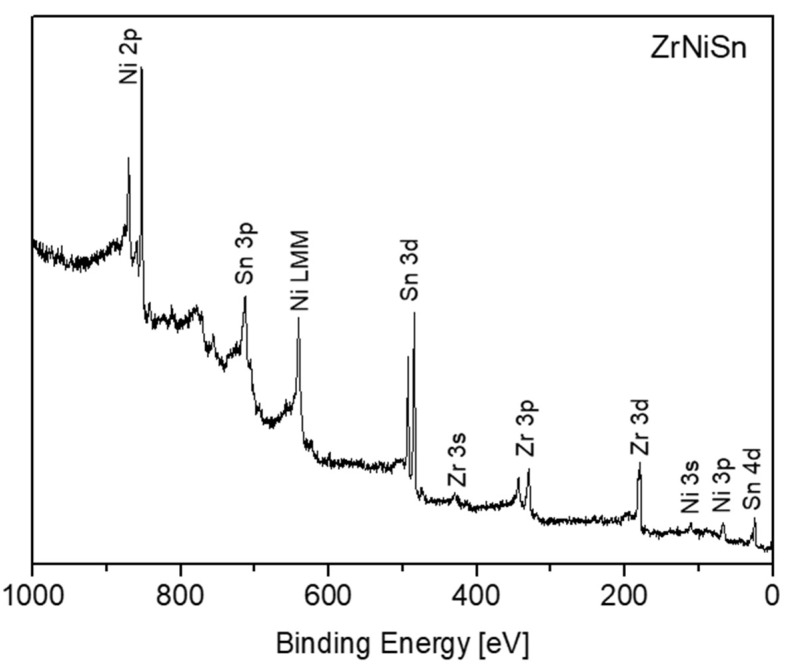
Survey spectrum taken from the ZrNiSn alloy surface after sputter-cleaning.

Survey XPS spectra of the ZrNiSn after sputter-cleaning at RT are presented in Figure 3. No measurable oxygen or carbon contamination could be observed. According to Graf et al. [3], in HH components having the general XYZ formula, the X and Y atoms are characterized by a cationic behavior, and the Z atom displays an anionic one. In the MNiSn family, where M denotes Ti, Zr or Hf, these elements and Ni are the cations, transferring charge towards the anionic Sn. This charge transfer tendency is expressed as chemical shifts of the elements in the alloy, compared to the known binding energy (BE) of the pure elements, as previously reported for the TiNiSn [11]. Table 1 depicts the measured BE of the elements from the clean ZrNiSn alloy. The values of the BE of the TiNiSn and the known common values of the pure elements from the literature are added for comparison. As can be seen, the Zr, Ti and Ni are shifted to higher BE, by 0.2–0.3 eV, while Sn is shifted to lower BE, in correspondence with the expected cationic and anionic behavior in the alloy.

**Figure 4:**
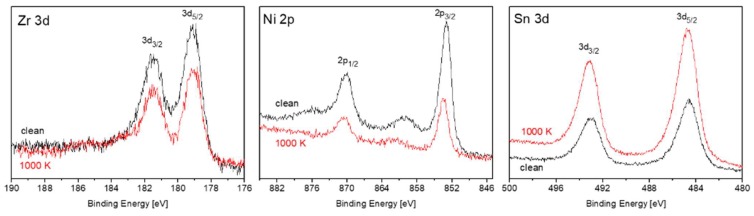
Zr, Ni and Sn spectra, taken from the ZrNiSn alloy before (black) and after (red) heating to 1000 K.

During our previous work on TiNiSn, it was found that heating the alloy in vacuum causes Sn segregation to the surface, and it was attributed to the considerably lower surface energy of Sn compared to the other components [11]. Since the surface energy of Sn (~0.7 J/m^2^) is considerably lower than the surface energy of Zr (~2 J/m^2^) and Ni (~2.4 J/M^2^) [17], it is expected that Sn will segregate to the surface, in the present case also. Figure 4 shows the Zr, Ni and Sn spectra, taken from the ZrNiSn alloy before and after heating to 1000 K. The attenuation of the Zr and Ni peaks and the growth of the Sn one indicate that Sn indeed segregates to the surface, as observed during heating of TiNiSn. Assuming that the Sn enriches the surface as a homogenous overlayer, its thickness can be evaluated from the intensity changes of the underlying elements, according to Equation (1) [18]:

*D* = − λ cos*θ* × ln(*I*/*I*_0_) (1)

where *d* is the overlayer thickness, λ is the inelastic mean free path (IMFP) of the electrons passing through the layer and *I* is the intensity of the underlying attenuated signal (the initial value is indicated by *I*_o_). *θ* is the angle between the analyzer entrance and the normal to surface (*θ* = 0°).

**Figure 5:**
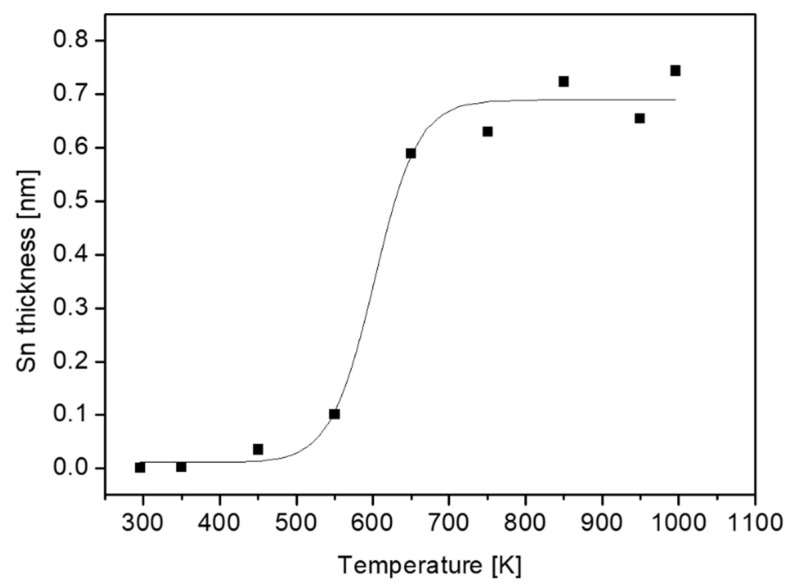
Sn layer thickness at the surface of the ZrNiSn alloy as a function of annealing temperature.

As can be seen from Equation (1), the layer thickness depends linearly on λ. However, there are no definite values for this parameter. IMFP, which is a function of electron energy and the material properties in which the electrons pass, is commonly evaluated by using the NIST database [19]. As for the previous report for TiNiSn, the results obtained from the predictive formula by Gries were adopted [11]. In order to follow the segregation dependence on temperature, the samples were heated to increasing temperatures for 15 min and monitored by XPS. The Sn layer thickness evaluated from Equation (1) by using the attenuation of the Zr signal by the Sn layer (λ = 2.1 nm) is depicted in Figure 5. Above 550 K, the segregation is vastly enhanced and the Sn overlayer reaches a thickness of about 0.7 nm.

### 3.2. Oxidation of ZrNiSn Alloy at Room Temperature

XPS spectra of the alloy components were recorded following oxygen exposure at 1 × 10^−6^ Torr (up to 50 L, the exposures were performed at 5 × 10^−7^ Torr). The thermodynamic driving force for oxidation is given by the Gibbs free energy difference during the reaction, which is given by ∆G = ∆H−T∆S (G is the Gibbs free energy, H the enthalpy, S the entropy and T the temperature). However, in our case, since the oxidation enthalpy is much higher than its entropy (3–4 orders of magnitude) [20], and the temperature range is limited, the entropy can be neglected. Hence it can be expected that the initial oxidation of the alloy components will follow the enthalpy of oxide formation, as for example in TiNiSn [11]. In the current study these enthalpies of oxide formation are [21]:

Zr + O_2_ → ZrO_2_, ∆H = −263 kcal/mol

Ni + ½O_2_ → NiO, ∆H = −57 kcal/mol

Sn + ½O_2_ → SnO, ∆H = −68 kcal/mol

The enthalpy of formation of the TiNiSn and ZrNiSn HH alloys were found to be ~−12.6 kcal/mol [22] and ~−16 kcal mol [23], respectively, remarkably higher than the oxidation enthalpies, and therefore the alloy is expected to oxidize. Furthermore, since Zr has the largest tendency to oxidize, it is expected that oxidation of the alloy will be governed by its oxidation. Zr has an oxidation state of 4 and therefore the formation of the dioxide is thermodynamically expected. 

**Figure 6:**
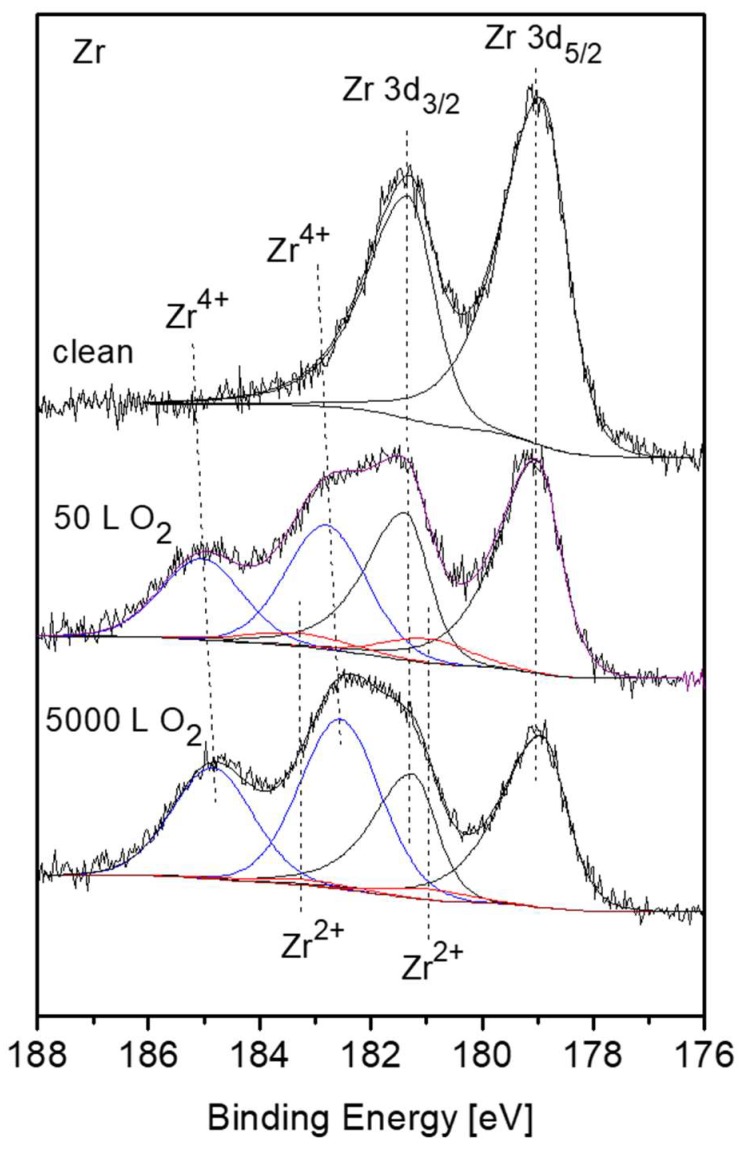
Representative XPS Zr 3d spectra, after different exposures at room temperature, with fitted metallic and oxide (Zr^4+^– blue, Zr^2+^– red) components.

**Figure 7:**
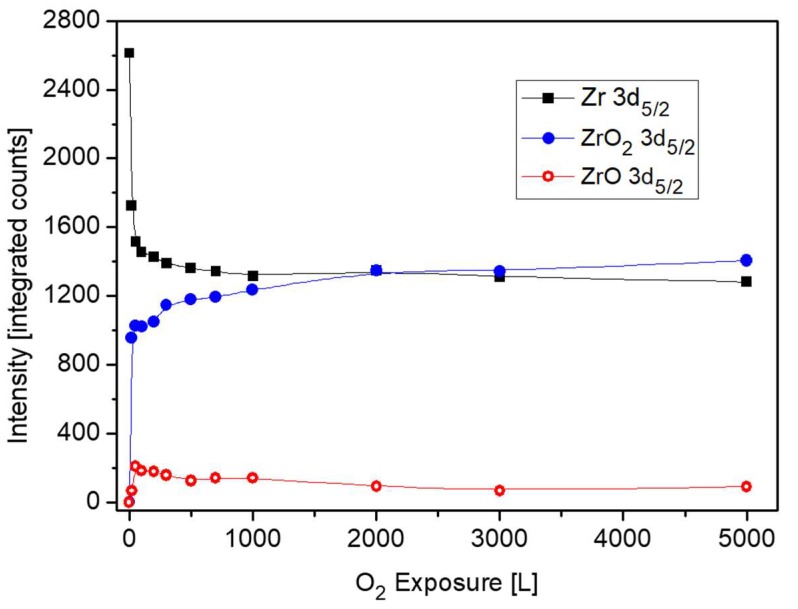
The metallic and oxides intensities of Zr vs. oxygen exposure at room temperature.

Nevertheless, there are some evidences in the literature that during initial oxidation of Zr metal at RT, a thin sub-oxide or defective oxide interlayer at the Zr-oxide interface, beneath the ZrO_2_ layer, is formed [24,25,26,27]. It must be emphasized that although attempts to fit up to three suboxides (Zr^+1^, Zr^+2^, Zr^+3^) are reported in the literature, due to the small separation of the peaks, their actual physical contribution to the spectra can be questionable, especially at high oxygen exposures. This is due to the limitation of instrumental resolution of conventional XPS equipment and the attenuation by the thicker ZrO_2_ overlayer. In order to gain better energy resolution and enhanced signal intensity, radiation from a synchrotron source was employed by Ma et al. [27] to investigate the early stages of Zr oxidation, and it was found that Zr^2+^ was the dominant sub oxide formed beneath the dioxide. The existence of ZrO as an interlayer at the metal-oxide interface was established for oxidation of Zr metal [28] and even Zr alloys [29]. Therefore, in the present data fitting of the XPS spectra, taken from the Zr component, the Zr^2+^ doublet with the 3d_5/2_ peak at BE of 180.99 eV [27] was incorporated, in addition to the ZrO_2_ and metallic peaks. The intensity ratio of the Zr 3d_5/2_ and 3d_3/2_ doublet, arising from spin-orbit splitting, was constrained to 3:2 for the metallic and oxidic components. Representative Zr 3d spectra with the fitted metallic and oxidic components are depicted in Figure 6, and the metallic and oxidic fractions, as extracted from the spectra, are shown in Figure 7. It can be seen that the Zr component in the alloy is readily oxidized at RT and Zr^4+^ (ZrO_2_) is the principal oxidation state. A small fraction of the oxide can be attributed to a sub-oxide in the form of ZrO or a Zr-rich defected layer at the interface. This sub-oxide is formed during the initial oxidation, reaching its maximum intensity at ~50 L and then its signal slowly attenuates by the growing of the ZrO_2_ overlayer.

**Figure 8:**
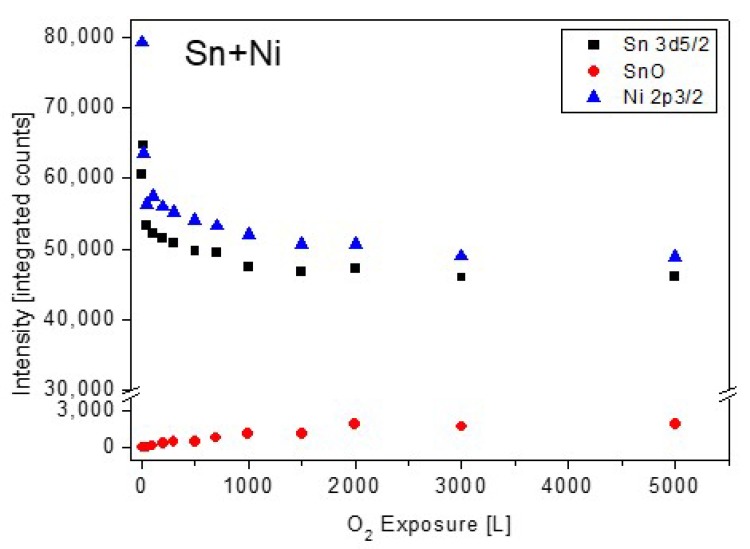
The metallic and oxides intensity of Sn and Ni vs. oxygen exposure at room temperature.

Analysis of the Sn and Ni spectra revealed that only very light oxidation of Sn can be seen at high exposures, with no oxidation signs of the Ni, similarly to the TiNiSn case (spectra not shown, see for similar results [11]). The attenuation of the Sn and Ni intensity during oxygen exposure are depicted in Figure 8.

**Figure 9:**
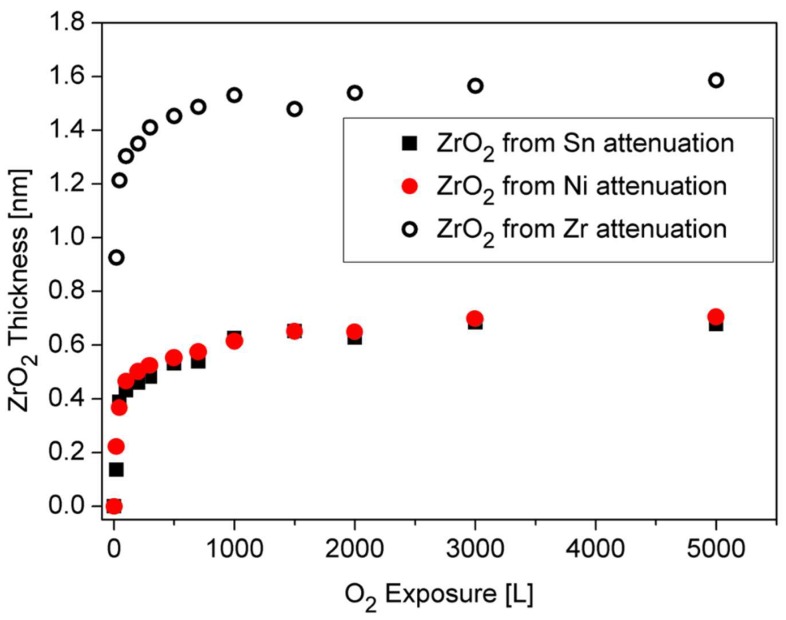
ZrO_2_ thickness formed on the surface during oxygen exposure at RT, as calculated from the attenuation of the Sn, Ni and Zr signals.

Observing the Sn and Ni lines shows that they were attenuated during oxidation. This attenuation implies the possibility that some oxygen-induced segregation of Zr to the surface occurs, and screens the Sn and Ni signals, as it is clearly seen at higher temperatures oxidation. The ZrO_2_ thickness formed at RT, which is shown in Figure 9, was estimated from the attenuation of the Zr, Ni and Sn peaks, using Equation (1). The IMFP of the Ni 2p_3/2_, Sn 3d_5/2_ and Zr 3d_5/2_ electrons passing through the ZrO_2_ film were evaluated as λ_Ni_ ≈ 1.4 nm, λ_Sn_ ≈ 2 nm [19] and λ_Zr_ ≈ 2.2 nm [25]. It can be seen that the oxide thickness obtained from the attenuation of Ni and Sn is similar (0.7 nm), while the oxide from the Zr line is much thicker (1.6 nm). These differences can point to the mechanism of the oxide formation. Since the Zr attenuation is influenced by the total thickness of the formed oxide, it can be deduced that the attenuation of the Ni and Sn peaks are governed by a fraction of the ZrO_2_ formed on top of the alloy. 

Therefore it can be argued that the oxidation occurs by oxygen-enhanced segregation of Zr to the surface to react with the oxygen (results in ~0.7 nm oxide) and by oxygen incorporation in the alloy and reacting with Zr to form the other ~0.9 nm oxide. 

### 3.3. The Interaction with O_2_ at 1000 K

**Figure 10:**
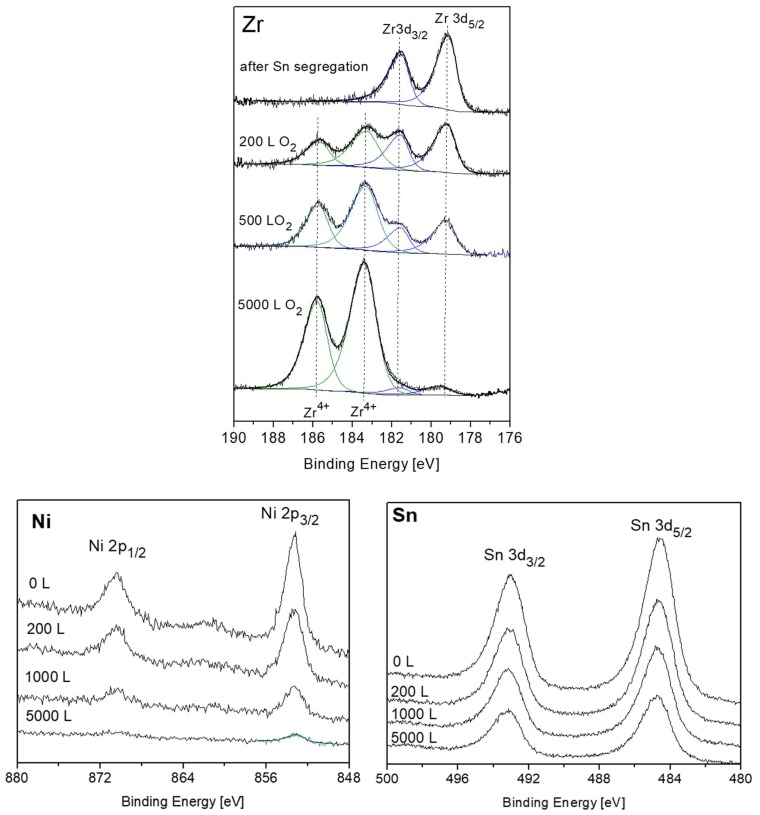
Representative XPS spectra of Zr, Ni and Sn after exposing to oxygen at 1000 K.

Previous to the exposure to oxygen at the elevated temperature, the sample was heated in vacuum at 1000 K for 15 min, to allow the Sn segregation to the surface. After each dose of oxygen, the oxygen was pumped out and the sample was cooled down prior to the XPS measurement. Representative XPS spectra of the alloy components during oxidation are presented in Figure 10. The spectra show rapid oxidation, almost disappearance of the metallic Zr signal at higher exposures and the formation of the Zr^4+^ oxidation state. During the fittings, the Zr^2+^ doublets were seemed to constantly cancel out, indicating that at the high temperatures this interlayer sub-oxide is not formed, or its amount is below the resolution or detection limit. Similar to RT, only minor oxidation of the Sn and no oxidation of the Ni can be seen, but the Sn signal attenuates significantly, and the Ni signal almost entirely disappears after 5000 L exposure. 

**Figure 11:**
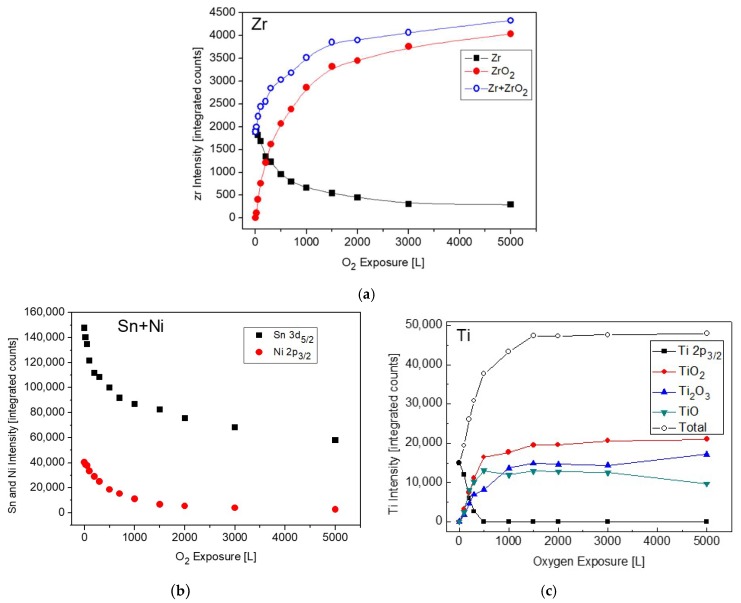
Intensity variation of: (**a**) Zr, ZrO_2_, (**b**) Ni and Sn and (**c**) Ti and its oxides, from [11] for comparison, during oxygen exposure at 1000 K.

The changes in the intensity of Zr, Ni and Sn during oxidation are depicted in Figure 11. For comparison, the Ti intensities during oxidation from [11], were also added (Figure 11c). Comparing the behavior of the Zr signals to those of the Ti ones, as was reported before for the case of the TiNiSn alloy [11], reveals some differences in the oxidation behavior. In the case of TiNiSn, the metallic Ti signal totally attenuated after ~500 L O_2_ exposure, but the total intensity of the Ti oxides markedly exceeds the intensity of the initial metallic signal. In addition, The Ni and Sn intensities were also totally attenuated at the higher oxygen exposures. This indicates that Ti readily segregates through the Sn overlayer (oxygen-enhanced segregation), and reacts at the surface with oxygen, forming an oxide of ~6 nm on the surface (see Figure 9 [11]). In the present case, the attenuation of the Zr is relatively moderate, and some metallic Zr peaks can still be seen even after 5000 L O_2_ exposure, in addition to the Sn signal. The small increase in the total Zr signal (Zr+ZrO_2_), compared to the initial Zr one, as seen in Figure 11, indicates that compared to the TiNiSn case, a smaller amount of Zr segregates through the Sn overlayer in order to oxidize. 

Since the crystal structure of the MNiSn (M = Ti, Zr, Hf) alloys ($F\bar{4}3m$) and the enthalpies of oxide formation are similar, it seems that the considerable differences in the atomic masses (Ti = 47.7, Zr = 91.2, Hf = 178.5 amu) controls the mobility of the atoms and the differences in the kinetics and mechanism of oxidation. 

**Figure 12:**
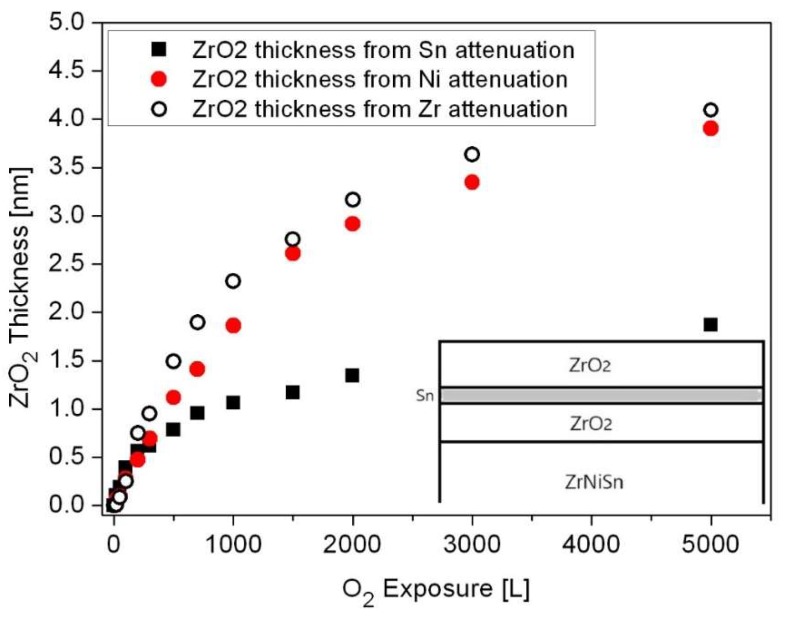
Oxide thickness formed during exposure to O_2_ at 1000 K, as calculated from the attenuation of the Sn, Ni and Zr peaks. The inset describes a schematic view of the oxide morphology.

The oxide thickness and the location where it forms (above or below the Sn layer) can be evaluated from the attenuation of the Sn, Ni and Zr signals, using Equation (1), as shown in Figure 12. Assuming the schematic view of the sample’s layers, shown as the inset in the figure, the attenuation of the Sn signal is caused by the ZrO_2_ formed on top of it, while the attenuation of the Ni and Zr intensities is governed by both the oxide formed at the alloy-Sn interface and the oxide formed on top. Embracing this relatively simplistic view indicates that the total ZrO_2_ oxide formed at 1000 K (after 5000 L exposure) is about 4nm, where a ~2 nm layer is formed beneath the Sn layer and a ~2 nm one above it.

### 3.4. Evaluation of the O 1s Spectra

**Figure 13:**
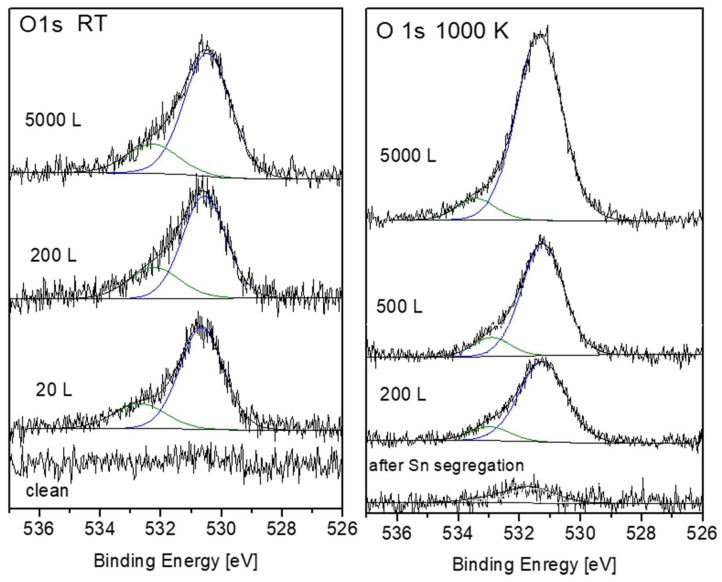
Representative O 1s spectra and peak fittings after oxygen exposure at RT and 1000 K.

**Figure 14:**
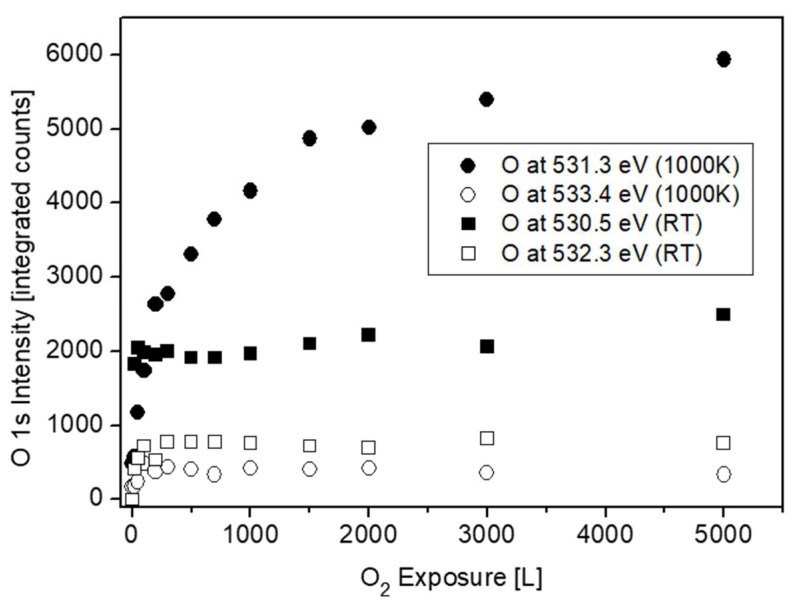
O 1s intensity vs. oxygen exposure at RT and 1000 K.

Representative O 1s spectra, collected from the surface after oxidation at RT and 1000 K, are shown in Figure 13. The spectra can be very reasonably fitted with two symmetrical mixed Gaussian-Lorentzian peaks (GL30). At RT, the dominant peak is located at BE = 530.5 ± 0.1 eV, followed by a weaker peak at higher BE = 532.3 ± 0.1 eV. At 1000 K, the main peak appears at BE = 531.3 ± 0.1 eV, with a much weaker peak at BE = 533.4 ± 0.1 eV. The intensities of these O 1s peaks are shown in Figure 14. While the main peaks are originated from the formed ZrO_2_, the origin of the high BE weaker peaks was questioned. During Zr metal oxidation, Lyapin et al [25] observed also similar high BE small peaks, which were attributed to adsorbed oxygen or hydroxides. Nevertheless, such peaks can also be attributed to a sub-oxide (as ZrO), or a Zr-rich oxide layer formed at the metal-oxide interface. The average composition of the main oxide was calculated from the O/Zr_(ZrO2)_, taking into account the relative sensitivity factors of O 1s (RSF = 2.93) and Zr 3d_5/2_ (RSF = 4.17), and was found to be slightly under stochiometric, O/Zr ≈ 1.8, in a good agreement with the composition found during Zr oxidation [24,25,30].

## 4. Summary and Conclusions

As part of the effort to characterize the surface and oxidation behavior of the MNiSn (M = Ti, Ni, Hf) half-Heusler alloys, in a continuation of a previous work on TiNiSn, the surface composition and oxidation behavior of the ZrNiSn alloy at RT and 1000 K is currently being reported. Comparing the binding energy of the components in the ZrNiSn alloy to those of the pure metals reveals that the Zr and Ni are shifted by ~0.2 eV to higher binding energy, while Sn is shifted to lower ones. These chemical shifts point on the charge transfer and the bonding nature of the semiconductor alloys, where Zr and Ni are cations, and Sn is an anion. During heating in vacuum, Sn segregates to the surface of the alloy, driven by the tendency to lower the surface energy, forming ~0.7 nm thick layer.

During the exposure to oxygen, it was found that the main oxidative element was Zr, accompanied by some minor oxidation of Sn. Careful data analysis of the Zr 3d and O 1s spectra revealed that most of the Zr is oxidized to the Zr^4+^ state (ZrO_1.8_), while at RT there is evidence to support the presence of a sub-oxide thin interface (probably in the form of ZrO) between the alloy and the main oxide. Whereas in the former case of TiNiSn, most of the oxide was formed by oxygen-induced segregation of the Ti and its oxidation at the surface, in the case of ZrNiSn, the mechanism is somewhat different. By calculating the oxide thickness from the attenuation of the components, it was deduced that part of the oxide is formed by Zr segregation to the surface, and part by oxygen dissolution into the alloy and oxidizing the Zr and some Sn. The thickness of the oxide layer, at 1000 K and after 5000 L O_2_ exposure, was evaluated as ~4 nm, about half of it formed above the Sn segregated layer, and the other half beneath it, by oxygen incorporation into the alloy and its oxidation. Future work will be held in order to characterize the surface and initial oxidation of the HfNiSn alloy. A study on the oxidation of these half-Heusler alloys at elevated temperatures and atmospheric pressure is under progress.

## Figures and Tables

**Table 1:** Binding energy (eV) of the elements in the alloys and the known literature values [12,13,14,16]. The chemical shifts are shown in brackets.

Xxx	Zr 3d5/2	Ti 2p3/2	Ni 2p3/2	Sn 3d5/2
literature	178.9	453.9	852.6	485
ZrNiSn	179.1 (+0.2)	–	852.8 (+0.2)	484.6 (−0.4)
TiNiSn	–	454.2 (+0.3)	852.9 (+0.3)	484.5 (−0.5)

## References

[1] Casper F., Graf T., Chadov S., Balke B., Felser C. (2012). Half-Heusler compounds: novel materials for energy and spintronic applications. Semicond. Sci. Technol..

[2] Rogl G., Grytsiv A., Gürth M., Tavassoli A., Ebner C., Wünschek A., Puchegger S., Soprunyuk V., Schranz W., Bauer E. (2016). Mechanical properties of half-Heusler alloys. Acta Mater..

[3] Graf T., Felser C., Parkin S.S.P. (2011). Simple rules for the understanding of Heusler compounds. Prog. Solid State Chem..

[4] Appel O., Schwall M., Mogilyansky D., Köhne M., Balke B., Gelbstein Y. (2013). Effects of microstructural evolution on the thermoelectric properties of spark-plasma-sintered Ti_0.3_Zr_0.35_Hf_0.35_NiSn half-Heusler compound. J. Electron. Mater..

[5] Appel O., Gelbstein Y. (2014). A Comparison Between the Effects of Sb and Bi Doping on the Thermoelectric Properties of the Ti_0.3_Zr_0.35_Hf_0.35_NiSn Half-Heusler Alloy. J. Electron. Mater..

[6] Appel O., Zilber T., Kalabukhov S., Beeri O., Gelbstein Y. (2015). Morphological effects on the thermoelectric properties of Ti_0.3_Zr_0.35_Hf_0.35_Ni_1+δ_Sn alloys following phase separation. J. Mater. Chem. C.

[7] Berry T., Fu C., Auffermann G., Fecher G.H., Schnelle W., Serrano-Sanchez F., Yue Y., Liang H., Felser C. (2017). Enhancing thermoelectric performance of TiNiSn half-Heusler compounds via modulation doping. Chem. Mater..

[8] Bankina V., Fedorova O., Leytus G. (1993). X-ray study of TiNiSn and ZrNiSn intermetallics oxidation. Mater. Sci. Forum..

[9] Gałązka K., Populoh S., Sagarna L., Karvonen L., Xie W., Beni A., Schmutz P., Hulliger J., Weidenkaff A. (2014). Phase formation, stability, and oxidation in (Ti, Zr, Hf) NiSn half-Heusler compounds. Phys. Status Solidi A.

[10] Berche A., Jund P. (2018). Oxidation of half-Heusler NiTiSn materials: Implications for thermoelectric applications. Intermetallics.

[11] Appel O., Cohen S., Beeri O., Shamir N., Gelbstein Y., Zalkind S. (2018). Surface Oxidation of TiNiSn (Half-Heusler) Alloy by Oxygen and Water Vapor. Materials.

[12] Biesinger M.C., Payne B.P., Lau L.W., Gerson A., Smart R.S.C. (2009). X-ray photoelectron spectroscopic chemical state quantification of mixed nickel metal, oxide and hydroxide systems. Surf. Interface Anal..

[13] Biesinger M.C., Lau L.W., Gerson A.R., Smart R.S.C. (2010). Resolving surface chemical states in XPS analysis of first row transition metals, oxides and hydroxides: Sc, Ti, V, Cu and Zn. Appl. Surf. Sci..

[14] X-ray Photoelectron Spectroscopy (XPS) Reference Pages. http://www.xpsfitting.com.

[15] Lu G., Bernasek S.L., Schwartz J. (2000). Oxidation of a polycrystalline titanium surface by oxygen and water. Surf. Sci..

[16] Moulder J., Stickle W., Sobol P., Bomben K. (1992). Handbook of X-Ray Photoelectron Spectroscopy.

[17] Vitos L., Ruban A., Skriver H.L., Kollar J. (1998). The surface energy of metals. Surf. Sci..

[18] Watts J.F., Wolstenholme J. (2003). An introduction to surface analysis by XPS and AES.

[19] Powell C.J., Jablonski A. (2010). NIST Electron Inelastic-Mean-Free-Path Database-Version 1.2.

[20] DoITPoMS university of Cambridge. https://www.doitpoms.ac.uk/tlplib/ellingham_diagrams/interactive.php.

[21] Weast R.C. (1970). Handbook of Chemistry and Physics.

[22] Gürth M., Grytsiv A., Vrestal J., Romaka V., Giester G., Bauer E., Rogl P. (2015). On the constitution and thermodynamic modelling of the system Ti–Ni–Sn. RSC Adv..

[23] Sauerschnig P., Grytsiv A., Vrestal J., Romaka V.V., Smetana B., Giester G., Bauer E., Rogl P. (2018). On the constitution and thermodynamic modelling of the system Zr-Ni-Sn. J. Alloy Compd..

[24] Lyapin A., Jeurgens L., Graat P., Mittemeijer E. (2004). The initial, thermal oxidation of zirconium at room temperature. J. Appl. Phys..

[25] Lyapin A., Jeurgens L., Mittemeijer E. (2005). Effect of temperature on the initial, thermal oxidation of zirconium. Acta Mater..

[26] Bespalov I., Datler M., Buhr S., Drachsel W., Rupprechter G., Suchorski Y. (2015). Initial stages of oxide formation on the Zr surface at low oxygen pressure: An in situ FIM and XPS study. Ultramicroscopy.

[27] Ma W., Herbert F.W., Senanayake S.D., Yildiz B. (2015). Non-equilibrium oxidation states of zirconium during early stages of metal oxidation. Appl. Phys. Lett..

[28] Nicholls R.J., Ni N., Lozano-Perez S., London A., McComb D.W., Nellist P.D., Grovenor C.R., Pickard C.J., Yates J.R. (2015). Crystal structure of the ZrO phase at zirconium/zirconium oxide interfaces. Adv. Eng. Mater..

[29] Dong Y., Motta A.T., Marquis E.A. (2013). Atom probe tomography study of alloying element distributions in Zr alloys and their oxides. J. Nucl. Mater..

[30] Morant C., Sanz J., Galan L., Soriano L., Rueda F. (1989). An XPS study of the interaction of oxygen with zirconium. Surf. Sci..

